# Short-term physical exercise impacts on the human holobiont obtained by a randomised intervention study

**DOI:** 10.1186/s12866-021-02214-1

**Published:** 2021-06-02

**Authors:** Lucas Moitinho-Silva, Michelle Wegener, Sandra May, Florian Schrinner, Awais Akhtar, Teide J. Boysen, Eva Schaeffer, Clint Hansen, Timo Schmidt, Malte C. Rühlemann, Matthias Hübenthal, Philipp Rausch, Mustafa T. Kondakci, Walter Maetzler, Stephan Weidinger, Matthias Laudes, Philip Süß, Dominik Schulte, Ralf Junker, Felix Sommer, Burkhard Weisser, Corinna Bang, Andre Franke

**Affiliations:** 1 Institute of Clinical Molecular Biology (IKMB), Christian-Albrechts-University of Kiel, Rosalind-Franklin-Str. 12, 24105 Kiel, Germany; 2 Department of Dermatology, Quincke Research Center, University Hospital Schleswig- Holstein, Kiel, Germany; 3 Institute of Sport Science, Christian-Albrechts-University of Kiel, Kiel, Germany; 4 Department of Neurology, University Hospital Schleswig-Holstein (UKSH), Kiel, Germany; 5 Department of Internal Medicine I, University Hospital Schleswig-Holstein (UKSH), Kiel, Germany; 6 Institute of Clinical Chemistry, Kiel University, Kiel, Germany

**Keywords:** Exercise, Intervention, Gut microbiota, Human health, Physiology

## Abstract

**Background:**

Human well-being has been linked to the composition and functional capacity of the intestinal microbiota. As regular exercise is known to improve human health, it is not surprising that exercise was previously described to positively modulate the gut microbiota, too. However, most previous studies mainly focused on either elite athletes or animal models. Thus, we conducted a randomised intervention study that focused on the effects of different types of training (endurance and strength) in previously physically inactive, healthy adults in comparison to controls that did not perform regular exercise. Overall study duration was ten weeks including six weeks of intervention period. In addition to 16S rRNA gene amplicon sequencing of longitudinally sampled faecal material of participants (six time points), detailed body composition measurements and analysis of blood samples (at baseline and after the intervention) were performed to obtain overall physiological changes within the intervention period. Activity tracker devices (wrist-band wearables) provided activity status and sleeping patterns of participants as well as exercise intensity and heart measurements.

**Results:**

Different biometric responses between endurance and strength activities were identified, such as a significant increase of lymphocytes and decrease of mean corpuscular haemoglobin concentration (MCHC) only within the strength intervention group. In the endurance group, we observed a significant reduction in hip circumference and an increase in physical working capacity (PWC). Though a large variation of microbiota changes were observed between individuals of the same group, we did not find specific collective alterations in the endurance nor the strength groups, arguing for microbiome variations specific to individuals, and therefore, were not captured in our analysis.

**Conclusions:**

We could show that different types of exercise have distinct but moderate effects on the overall physiology of humans and very distinct microbial changes in the gut. The observed overall changes during the intervention highlight the importance of physical activity on well-being. Future studies should investigate the effect of exercise on a longer timescale, investigate different training intensities and consider high-resolution shotgun metagenomics technology.

**Trial registration:**

DRKS, DRKS00015873. Registered 12 December 2018; Retrospectively registered.

**Supplementary Information:**

The online version contains supplementary material available at 10.1186/s12866-021-02214-1.

## Background

Due to its exposure to the external environment, the human intestine harbors a plethora of microbes forming a complex ecological community [1, 2]. Most of these microbes are not only crucially involved in digestion and fermentation processes, but also exhibit protective and structural functions for their host [1–3]. With respect to this, the stability and individuality of human gut microbiomes have been shown to change with age, lifestyle and diet, but are also affected by the host’s genotype [4, 5]. However, in contrast to the mostly static human genome, the microbiome composition is more dynamic and has been found to be altered in patients of different diseases [6]. Overall, numerous studies demonstrated that a healthy host-microorganism balance appears to be crucial to maintain optimal metabolic and immune functions as well as prevention of disease development [7]. Consequently, the microbiome is discussed as a potential target in precision medicine [8], though this goal still needs not only a mechanistic understanding of the known associations but also accurately targeted and safe intervention methods.

With regard to interventions, the positive influence of physical activity on the musculoskeletal, the nervous, the cardiovascular, the respiratory and the hormone system as well as the underlying mechanisms has already been described in detail (reviewed in [9, 10]). While the so far defined effects on the skeletal muscle system mainly depend on morphological and functional adaptations, impacts on internal organs and on the cardiovascular system appear to be mainly driven by metabolic, neuronal and hormonal alterations obtained by steady physical activity [11, 12]. Notably, after a period of regular training, the parasympathetic components of the nervous system have been shown to become predominant over sympathetic ones, thus resulting in better regeneration, improved mental balance and an overall economization of the metabolism [13]. An improved understanding of the underlying neurobiological mechanisms could be crucial to develop preventative or therapeutic intervention strategies for neurological disorders such as Alzheimer’s disease and depression further, since there has been an increasing recognition of metabolic dysfunction in these diseases [14]. With respect to this aim, the extended research during the last years has identified the gut microbiome as one of the key players in the gut-brain-axis, thereby linking the research fields for physical activity, neurology and microbiota (reviewed in [15]). To this end, several studies already addressed the question if and how exercise may affect the gut microbiota or *vice versa* (very recently reviewed in [16]). Though most related studies focused on animal models, there are also some intervention trials as well as cross-sectional and observational comparisons in humans. However, the participants that underwent the interventions were rarely healthy adults (detailed summary of the previous studies in [16]). In summary, cross-sectional studies on the effect of regular exercise on the gut microbiota mainly revealed an increased relative abundance of butyrate-producing taxa (e.g. *Faecalibacterium prausnitzii* and *Roseburia hominis*) [17, 18] as well as a higher abundance of *Akkermansia muciniphila*, a bacterial species that has been linked to improved metabolic health and a reduced body mass index (BMI) [19]. These findings were partially confirmed in longitudinal studies, though the effects were found to be reversed after a subsequent sedentary period and thus demonstrating its transience [20, 21].

In general, current research strongly supports the hypothesis of exercise being an lifestyle factor that can alter the gut microbial composition with beneficial effects on the host [22]. This finding might be implicated in the overall concept of precision medicine. However, a more detailed knowledge on the molecular crosstalk between the host and its microbiota is necessary. To further deepen our overall understanding of exercise effects on the human host, we initiated the herein presented randomized controlled intervention trial that focused on healthy individuals. Extensive data on biological samples, health status and physical activity before, during and after different intervention strategies were collected and analysed. Though the intervention period was only six weeks — mainly to be able to compare the results to earlier studies [23] — we observed trends in human response when comparing strength and endurance training groups, strongly arguing towards personalized intervention strategies in the future.

## Results

**Fig. 1:**
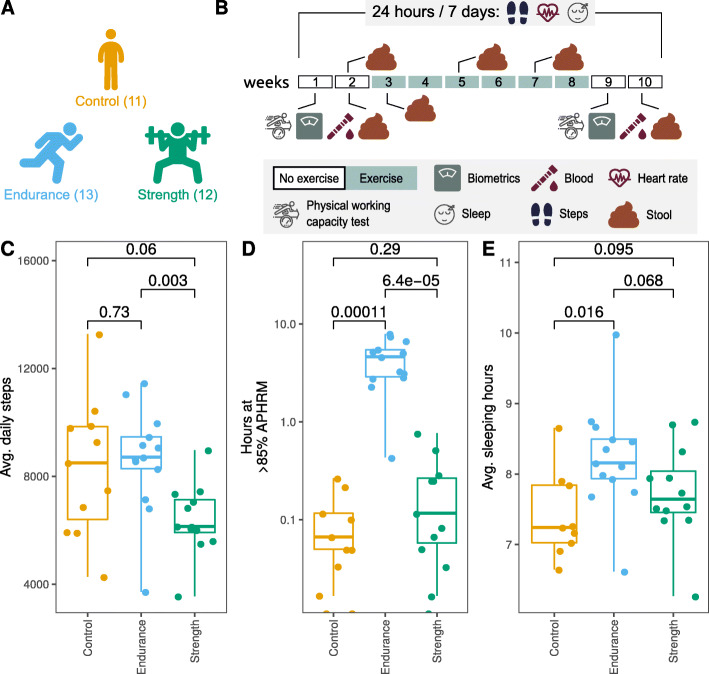
Graphical summary of study design and participants’ behaviour. **a** Number of included participants of the different groups are shown in parentheses. **b** Exercise intervention duration and sampling timepoints are shown. Activity tracker data (24 hours/7 days) were obtained from GARMIN® wristband devices. **c** Participants’ average daily steps, **d** summed hours at > 85 % age-predicted maximum heart rate (APHRM) and **e** average sleeping hours are represented. *P*-values for comparisons between groups are shown

During the presented randomized controlled intervention trial, 11 controls, 13 participants of the endurance group and 12 participants of the strength group completed the whole program (Fig. 1 a). Only data from these participants (including body composition measurements, analysis of blood samples, microbial analyses of faecal material of participants as well as information by tracker devices, Fig. 1b) were included in the following analyses. Main dropout reasons were non-compliance (*n* = 5) and antibiotic use during the intervention (*n* = 1).

All data from this study was loaded and integrated into a data warehouse with an interactive browser as a front-end (for detailed description see Methods section). This browser was used to continuously test for completeness of data (and so compliance of users) and for implementation of data mining algorithms to perform explorative analyses of the groups and group comparisons.

### Observations obtained from tracker devices

Remarkable variations were observed between the groups’ walking, use of aerobic capacity and sleeping averages considering the whole measured interval. Participants of the group endurance (8624 ± 1971) had significantly higher (*P* value 0.003) average daily steps in comparison with participants of the group strength (6394 ± 1320) (Fig. 1 c). Unexpectedly, average daily steps of control participants (8332 ± 2554) were very similar (*P* value 0.73) to participants of endurance (8624 ± 1971). Similarly, participants in the endurance group had statistically significant higher summed hours at > 85 % age-predicted maximum heart rate (APHRM) (4.41 ± 2.2) in comparison with strength (0.2 ± 0.23, *P* value < 0.001) and control (0.08 ± 0.09, *P* value < 0.001) groups (Fig. 1d). In addition, participants of the group endurance had more sleeping hours than controls (*P* value 0.016, control: 7.41 ± 0.62, endurance: 8.23 ± 0.76, strength: 7.74 ± 0.67) (Fig. 1e). Intra-group variation in daily steps and sleeping hours were further tested. No significant variation was found in the first. A slight decrease in sleep length between the beginning of measures and the end of the intervention was found in participants of the strength training group (Suppl. Figures 1 and 2).

### Change in body measurements, physical capacity, and blood profile

**Fig. 2:**
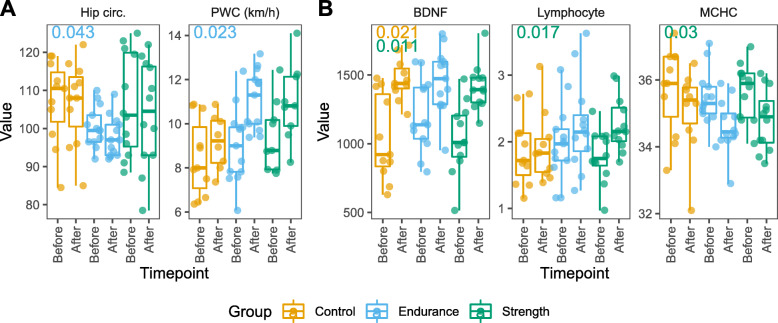
Body measures and blood profile change before and after the exercise intervention period. Within-group changes in **a** body measures and **b** blood profiles were tested separately. Direction changes were similar for all groups for the measures shown. Adjusted P values of significant results (*P* < 0.05) are shown for each group in the upper left diagram corners for *After* versus *Before* comparison. Hip circ. = Hip circumference; PWC = physical working capacity 170 test; BDNF = brain-derived neurotrophic factor; Lymphocyte = lymphocyte counts x10^9^/l; MCHC = mean corpuscular hemoglobin concentration

Changes in biometric measurements, physical capacity, and blood profile were tested within the three groups (Fig. 2). We detected a significant reduction in hip circumference and increase in physical working capacity (PWC) in participants after endurance training. Three physiological markers measured in blood showed significant changes after the intervention period. The neurotrophic brain-derived neurotrophic factor (BDNF) was significantly increased in participants of the control and strength groups. In addition, the lymphocyte numbers significantly increased and mean corpuscular haemoglobin concentration (MCHC) decreased in participants of strength group. Noteworthy, the direction of change of the mentioned metrics was similar among groups. The summary of biometric and blood profile tests can be visualized using our prepared data freeze and interactive browser (see above and Suppl. Figures 3 and 4).

### Microbial and dietary changes during the intervention

**Fig. 3:**
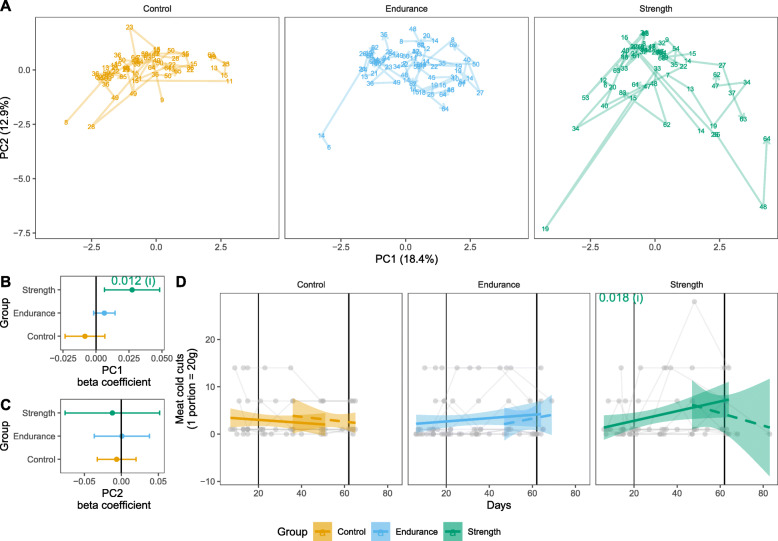
Participant’s dietary habits. Dietary components or participants were summarized using principal component analysis (**a**, PCA). Each plotted number represent the dietary pattern of the stool sampling date summarized into two dimensions. Dietary information refers to the precedent week of the sampling day. Participants’ data are connected by line. Two scenarios were modelled to test for (i) within group differences in averages before and during the exercise intervention period (solid line) and to test for (ii) within group differences in averages after the exercise intervention (dashed lines). These models were applied to PC1 to test for the effect of days (β coefficient) on the general dietary habit of participants (**b** and **c**). Solid and dashed lines refer to the two model scenarios. Intake of meat cold cuts was increased in participants of strength group during intervention period (**d**)

Within group changes in diet and microbiome were tested to answer two questions: (i) “Is there a change from the start of the study until the end of the physical activity intervention?” and (ii) “Is there a change towards the end of the intervention period and after it?” (see Materials and Methods for further details). General patterns of the diet information of the week prior collection of stool samples were summarized by PCA. The PC1, which explained 18.4 % of diet variation was tested to answer the two questions above (Fig. 3 a). There was a significant change in dietary pattern of participants of the strength group during physical intervention (Fig. 3b). This change may be, at least partially, driven by a significantly increased ingestion of cold meats in the participants of the strength group during intervention (Fig. 3d). No other dietary component intake changed significantly during the intervention period (Suppl. Figure 5). No change was found in dietary patterns and component intakes in participants of the control and endurance groups (Fig. 3 c, Suppl. Figure 5). Testing for question ii produced no significant results. To acknowledge for dietary effects on the participant’s microbiome, the first PC dimension was included in the statistical models applied to the microbiome data.

**Fig. 4:**
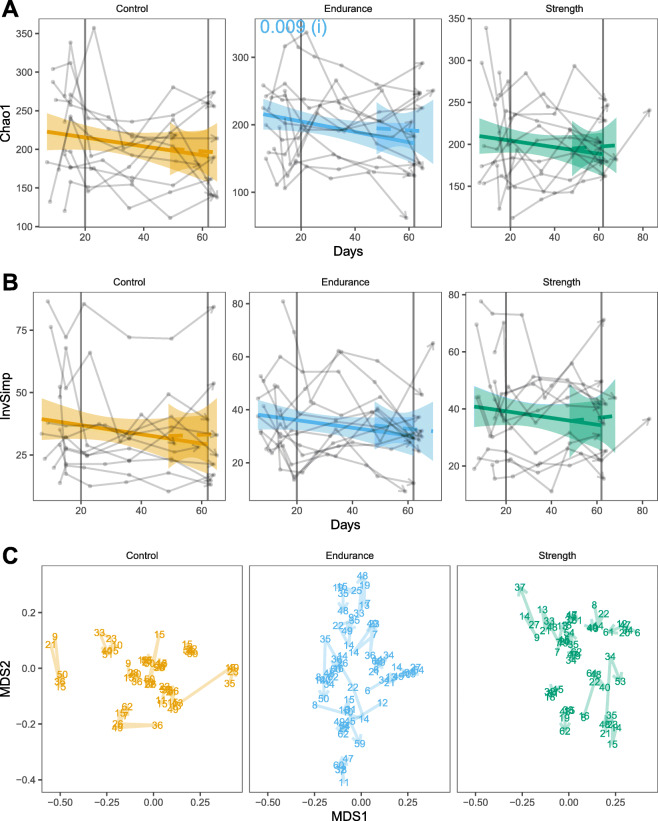
Changes in the gut microbiome over time of different groups. Alpha diversity estimation of richness (**a**, Chao1) and diversity (**b**, Inverse Simpson - InvSimp) are shown. Two scenarios were modelled to test for (i) within group differences in averages before and during the exercise intervention period (solid line) and to test for (ii) within group differences in averages after the exercise intervention (dashed lines). Vertical lines indicate the beginning and end of the exercise intervention interval. P values of significant results (*P* < 0.05) are shown. **c** Nonmetric multidimensional scaling (NMDS) was used to visualize beta-diversity, i.e. samples’ Bray-Curtis dissimilarities. Numbers represent sampling days. The stress of the NMDS plots was 0.28, indicating a poor goodness of fit

Changes of the microbial communities were analysed from four different perspectives: alpha diversity (Chao1 species richness and Inverse Simpson index), beta diversity (Bray-Curtis dissimilarity), changes in bacterial abundance and changes in bacterial occurrences (Fig. 4). With regards to variation in diversity metrics for analysis (i), there was a reduction in the estimated number of different bacterial taxa (amplicon sequence variants - ASVs) per sample (Chao1, P value 0.009, Fig. 4 a) in the endurance group. No significant change in estimated richness (Chao1) was observed in the control and strength groups. None of the groups showed any response to the intervention with respect to community diversity (inverse Simpson), nor community structure (Bray-Curtis) (Fig. 4b c). Likewise, there was no significant variation in the abundance neither occurrence of ASVs within groups.

### Microbiome differences between elite athletes and physically inactive participants

**Fig. 5:**
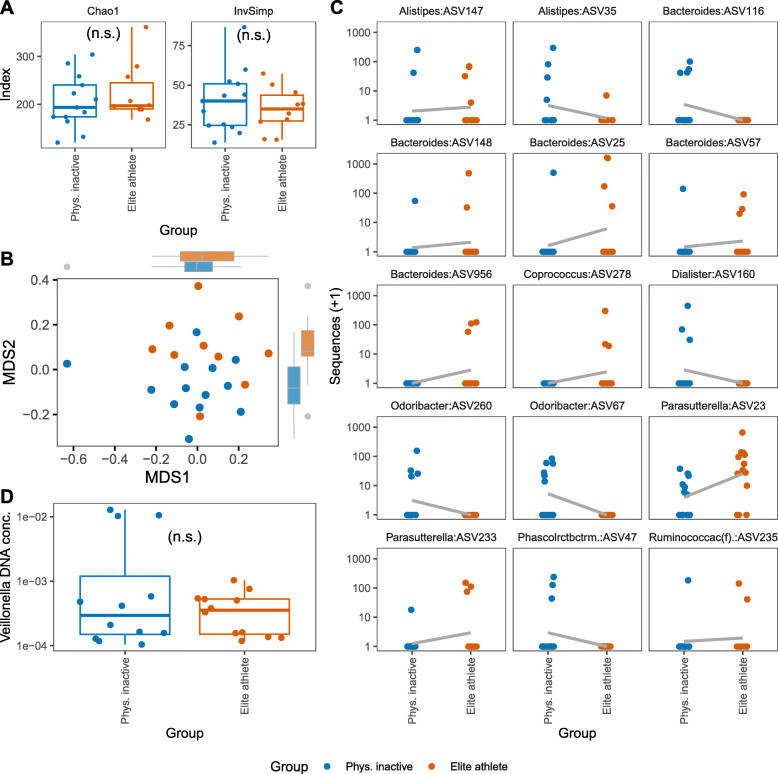
Comparison of gut microbiome in physically inactive participants vs. elite athletes. **a** Alpha diversity estimation of richness (Chao1) and diversity (InvSimp) are shown. **b** Nonmetric multidimensional scaling was used to visualize beta-diversity, i.e. samples’ Bray-Curtis dissimilarities. The stress of the NMDS plots was 0.26, indicating a poor goodness of fit. Marginal boxplots were added to visualize distribution of samples along axis. **c** ASVs found to be statistically more abundant in physically inactive participants or in elite athletes (adjusted *P* values < 0.05). Names of the bacteria genera are shown. *Phascolarctobacterium* and *Ruminococcaceae* (ASV classified to the family level) were abbreviated. A regression line was fit to illustrate the direction of change. **d** DNA concentration from *Veillonella* bacteria were quantified by qPCR using microbial DNA extracted from stool. No statistically significant difference was found for *Veillonella* between physically inactive participants and elite athletes

In search for microbiota associations with physical training, we compared the microbiome of elite athletes and the physically inactive participants. Physically inactive participants were of the same sex and of similar age as the elite athletes. No statistical differences in alpha diversity measures (Chao1 and Inverse Simpson index) or beta diversity (Bray-Curtis dissimilarity of ASV abundances) were observed (Fig. 5 a and b), despite the differences of sample distributions evident from visual inspection of the non-metric multidimensional scaling plot (Fig. 5b). A few ASVs belonging to the genera *Coprococcus*, *Parasutterella* and to the family *Ruminococcaceae* were found to be significantly more abundant in elite athletes in comparison to physically inactive participants (Fig. 5 c). On the other hand, ASVs belonging to the genera *Dialister, Odoribacter*, and *Phascolarctobacterium* were found to be more abundant in physically inactive participants as compared to elite athletes. Finally, various ASVs from the genera *Alistipes* and *Bacteroides* were found enriched in physically inactive participants and elite athletes with four *Bacteroides* ASVs being exclusively enriched in elite athletes. Furthermore, there was no significant differences in the occurrences of ASVs between the two groups.

Because members of the genus *Veillonella* were previously reported to be associated with elite athlete’s microbiome (detected in higher significances in two independent cohorts after competition [24], we decided to compare the abundance of members of this genus in physically inactive participants and elite athletes (*n* = 12; qPCR for one participant failed). *Veillonella*-specific qPCR DNA quantification was used to determine absolute cell numbers of *V. atypica* in stool DNA samples of both groups. *Veillonella* abundances measured via *Veillonella* (i.e. *V. atypica*) specific qPCR or via sequencing (ASV abundance) did not differ significantly between physically inactive participants and elite athletes.

## Discussion

The overall aim of this study was to examine the short-term effects of different types of structured physical exercise on the human gut microbiota in previously rather sedentary healthy German adults. To ensure the overall understanding of exercise effects on the human host, extensive data on biological samples, health status and physical activity before, during and after different intervention strategies were collected and analysed.

Both the endurance as well as the strength interventions were successful in achieving systemic effects on participants as endurance group achieved higher PWC scores after the intervention and blood cell profile in participants of strength group were altered. The PWC score was generated through running, which may explain the better performance of the endurance (running) group. Although changes in body composition were mostly non-significant, they showed a tendency towards a reduction in fat mass in the endurance and strength group. In addition, there was a small but significant reduction in hip circumference in the endurance group, though the training program in the present study was not primarily designed to produce significant changes in this parameter. These results underline the benefit of a sport intervention.

The blood cell profile before and after the intervention in both, the endurance and the strength group, revealed similar “positive health trends” for most changes. On the one hand, moderate decreases of MCHC, MCH and normoblasts argue for a general effect of physical activity on the enhanced oxygen delivery and its availability to the tissues – though even in elite triathletes, long-term endurance training did not largely alter haematological status [25]. On the other hand, slight increases in monocytes, lymphocytes and leukocytes may hint at regulatory mechanisms involved in the immune response for muscle repair after exercise intervention as has been shown before [25]. Our findings of a significant increase of lymphocytes levels exclusively after 6-weeks of strength intervention as well as the observed increase in muscle mass in this group underline the first acute pro-inflammatory effects that have been observed earlier in sedentary healthy adults after starting strength training protocols [26]. Improving low-grade inflammation as a long-term effect is considered to be an effective way to prevent and delay chronic inflammatory diseases [27].

Our microbiome analyses revealed a slight reduction in the number of different taxa found in endurance participants. Such microbiome modulation is not satisfactorily explained by the apparent increase in white blood cells in endurance participants, albeit not significant (Supplementary Fig. 3). Otherwise, a significant reduction in the number of different taxa would have been found in participants of the strength group, which showed elevated lymphocytes counts in blood after the intervention period and whose blood cell profiles were increased after the intervention period. At the end of intervention, endurance training participants accumulated longer periods of high circulatory system usage (Fig. 1) in comparison with participants performing strength training. As a result of strenuous physical exercise, blood flow is redirected to active muscles, cardiopulmonary system and skin, leading to reduction of gastrointestinal blood flow [28, 29]. Therefore, we hypothesize that acute physiological changes of the gastrointestinal environment caused by the reduction of gastrointestinal blood flow during exercise, such as mucosal damage and inflammation [30], may be one of the mechanisms involved in the modulation of microbiome richness in individuals under short-term endurance training. We did not find specific bacteria that varied in the endurance nor the strength groups. It is possible that microbiome variation due to physical exercise intervention is specific to individuals, and therefore, collective alterations were not captured in our analysis. In support to this hypothesis, we observed that microbiome change patterns largely varied between individuals of the same group (Fig. 4, e.g. Suppl. Figure 8). Future studies with more focus on individual-wise microbiome changes are therefore suggested.

To elucidate if potential structural differences between the microbiota of physically inactive individuals and elite athletes may exist, and to validate previous findings of other groups, we analysed stool samples from age- and sex-matched participants of these groups. Unexpectedly, no significant differences were observed in microbial alpha- and beta-diversities between these groups. Previous studies, however, detected a slight increase of diversity within elite athletes, though these were mostly professional athletes such as ultra-endurance rowers, competitive cyclists, marathon runners or rugby players [24, 31–33]. In line with these studies, we found higher abundances of various *Bacteroides* species in elite athletes probably explained by an elevated protein intake. However, all differentially abundant species were only found at low abundance levels, thus these results should be interpreted with care and rather as minimal differences rather than community-wise structural changes. Besides, no dietary data for elite athletes were available to acknowledge possible confounding effects when compared to physically inactive participants. In addition, abundance of *V. atypica* was investigated, since this specific species has been shown to enhance athletic performance (specifically found in marathon runners post competition) by its metabolic conversion of exercise-induced lactate into propionate [24]. However, only marginal differences were observed in a subset of marathon runners and other subsequent studies failed to detect an increased abundance of *Veillonella* species in elite athletes [31].

A main limitation of our study is the control group behaviour. While participants were asked at the beginning of the study to keep their sedentary lifestyle, the control group had similar values in average of daily steps when compared to the endurance group and even higher values when compared to the strength group during the entire study. So, either the control persons were overestimating their sedentary lifestyle before the study or study participation and wearing a wrist-band fitness tracker motivated them (unconsciously) to increase daily activity. However, the high number of average daily steps in the control group were not due to demanding physical activity as indicated by their low cumulated hours at high heart rate. Therefore, the control group could be considered as performing low intensity physical activity. As most of the participants were University students and as this study started right at the beginning of the winter semester, our observation may mainly reflect the daily routine of students within the semester, including physical activities such as biking back and forth to the University or walking to lecture halls on the rather extensive campus, for example. The observed increase of BDNF in the control group, which is comparable to the strength group, ratifies the practice of low intensity physical exercise of the first. Serum BDNF levels have been shown to increase after different kinds and intensities of exercise in healthy individuals [34–37]. Interestingly, BDNF increase has been previously associated with a positive impact on cognitive function and memory performance [25, 38]. With respect to the measured sleeping hours of participants, we could not detect any significant differences that could be related to the sport intervention. This finding is in line with other studies [39]. Therefore, this factor was considered constant, and not controlled for, in our statistical analysis.

## Conclusions

In summary, by combining detailed body composition measurements, analysis of blood samples, microbial analyses of faecal material of participants as well as information from tracker devices, we could show that different types of exercise have diverse effects on the overall physiology of individuals. Any changes observed during this (already relatively short) intervention time appear to improve human health, thus underlining the importance of physical activity on human health, counteracting disease-related processes. Compliance for trackers was very good and collected data was helpful in our analyses. Future studies should employ more validated and accurate wearable devices (lack of benchmarking data in community so far), aim at a longer intervention period, investigate different exercise intensities, and use more high-resolution and quantitative (but more expensive) metagenomic shotgun Next-Generation-Sequencing (NGS) technologies. It may also be important to collect 9–12 samples as baseline to get a better quantitative estimate of individual bacteria and their dynamics [40]. Finally, the effects of different exercise may be very individual, arguing for personalized training programs.

## Methods

### Ethics

The study was conducted in accordance with the Declaration of Helsinki and adheres to CONSORT guidelines (http://www.consort-statement.org/). Ethical approval was granted by the ethics committee at Kiel University (D534/18). All volunteers provided written informed consent.

### Study design and cohort sampling

For the presented randomized controlled intervention trial (Trial registration: DRKS, DRKS00015873. Registered 12 December 2018 - Retrospectively registered, https://www.drks.de/drks_web/navigate.do?navigationId=trial.HTML&TRIAL_ID=DRKS00015873), 42 healthy physically inactive German male and female volunteers aged 20 to 45 years and with a body mass index (BMI) between 20 and 35 kg/m^2^ were recruited for strength, endurance or control group participation (Fig. 1 a) in October 2018. Exclusion criteria comprised regular medication intake, the intake of antibiotics six weeks prior to the study, known existing chronic diseases and regular exercise during six months prior to the study. The overall study duration was ten weeks (Fig. 1b). During the first week, participants came into the study centre for a comprehensive baseline assessment, comprising measurements of height, weight, blood pressure, pulse, waist to hip-ratio, hip circumference, and body composition. In addition, blood samples were taken and wrist-band fitness trackers with dedicated smartphones were provided to the study participants. Subsequently, endurance levels were measured by a physical working capacity 170 (PWC) test. During the study period, participants were asked for six stool samples collected at home, sending to the laboratory immediately (for detailed sampling see Fig. 1b) and to fill in questionnaires for each of the stool samples regarding their health and diet the week before the sample was taken. Participants longitudinal data was synchronized so the intervention starts at day 20.

To study the effects of different type of exercise, participants were randomly divided into three groups, namely control, endurance, and strength, matching for gender, age, and BMI. Participants of the control group were asked to maintain their general physical inactivity. However, a guided exercise program after the end of the study was offered to the control group as an incentive. During the six weeks long intervention, participants of the endurance group were required to run three times per week for at least 30 min. One session per week was supervised by a professional trainer. The intensity of the run was regulated by the usage of the Borg rating of perceived exertion (RPE), representing a “basic” endurance training. Participants of the strength group performed a whole-body hypertrophy strength training in the gym three times a week, of which two trainings per week were supervised. In brief, the participants had a five-minute warm-up on the treadmill, ergometer, or rowing machine before they started their training of approximately 30 min. One session consisted of six different exercises, each two for the legs, the chest, and the back to train the large and main muscle groups. The participants performed one warm-up set (supposed to be 50 % of the load set weight) and one load set for every exercise. For the load set, the weight was chosen to ensure eight possible repetitions. If more than eight repetitions were possible on two consecutive training days, the participants were required to raise the weight in the following session. Both groups were asked to fill in a detailed training journal during the unsupervised training sets to ensure training intensity.

During the last week of the study (week 9), again assessments of height, weight, blood pressure, pulse, waist to hip-ratio, hip circumference and body composition were made for all participants. Blood samples were taken and finally, endurance level was measured by PWC test (Fig. 1b).

In addition to the physically inactive volunteers of the intervention study, and for generating a data set from an “extreme exercise” group for comparison, 13 elite athletes (mainly cyclist and triathletes) were each asked to provide faecal samples. Table 1 summarizes the demographic and anthropometric characteristics of the study participants at the beginning of the study as well as the respective information on elite athletes.

**Table 1:** Baseline demographics and anthropometric data of the study participants

Group	n	Sex (%m/%f)	Age	Weight (kg)	BMI	PWC^a^ (km/h)
Control	11	36/64	33.4 ± 7.9	78.7 ± 18.2	26.9 ± 5.6	8.5 ± 1.7
Endurance	13	23/77	31.4 ± 8.3	68 ± 10	23.1 ± 3.2	9 ± 1.7
Strength	12	50/50	29.9 ± 7.9	85.6 ± 25.7	26.3 ± 6.6	9.4 ± 1.6
Elite	13	38/62	30 ± 9.9	-	-	166.8 ± 159.9

Average (∅) values are shown and standard deviation is depicted as ±

^a^ Physical working capacity 170 test

### Measurement of blood analytes

Blood samples taken from each participant at the beginning and the end of the study period were analysed by complete blood counts at the Institute of Clinical Chemistry, UKSH Kiel, Kiel, Germany. Additionally, serum samples were used to measure blood concentrations of brain derived neurotrophic factor (BDNF) by using a commercially available Human BDNF ELISA Kit (Sigma-Aldrich, St. Louis, Missouri, US) at the end of the study. The full set of blood measurements is displayed in Suppl. Figure 3. Serum samples were diluted 200-fold and ELISA was performed according to the manufacturers protocol.

### Processing of data collected by the fitness trackers

Generally, raw data collected by current consumer-grade fitness trackers comprises movement intensities, step counts, heart rate, heart rate variation and, if implemented, additional data like activity types, such as sleep-related information. These data sets are forwarded by a corresponding application on the smart phone to servers operated by the vendor. Here data aggregation and the derivation of further health-related values like stress scores or sleep phase detection takes place. This processed data is used by the applications to illustrate a health and fitness status to the users.

In preparation for this study, several consumer-grade fitness trackers were tested for data accuracy and accessibility especially regarding step counts and heart rate readings. The accuracy of the step counts was tested both with repeated walking tests with defined step counts, by bicycling rounds and reproducing various everyday situations to test for correct and false step counts, respectively. Heart rate detection was verified with medical heart rate monitors. After the decision for a suitable tracker, the infrastructure needed to receive the data was developed and tested. Data validation included the analysis of raw data on the tracker to ensure data integrity and statistical adequacy. In this study the GARMIN® Vívosport fitness tracker was used. The data was collected and uploaded by the GARMIN® Connect application on the provided smartphone equipped with a pseudonymized user account. To transfer the data in our research data warehouse, GARMIN® Health API Endpoints were developed (https://developer.garmin.com/health-api/overview/). To verify the data integrity, the raw data was extracted from the device and verified with the data retrieved with the Health API. During and after the study, the complete dataset was verified for possible errors and artifacts. Only values proven to be reliable in the data validation — steps, heart rate and sleep lengths — were used in this study. Sleep length data from participants with less than 10 days of reliable and complete sleep length information were disregarded. Age predicted maximum heart rate (APHRM) was calculated using the formula by Tanaka et al. [41]. Finally, the data was moved into a relational database schema for processing and to make it available both for interactive browser applications and statistical analysis.

### Interactive Web Portal

Due to longitudinal nature of the data, an interactive web portal was developed using Springboot and highcharts libraries to visualize this data. The main goal of this portal was to aid data compliance for each individual participant. Study manager could login to view pseudonym participants, their group, and accompanying data. Suppl. Figure 6 presents such data as timeline which allows study manager to have quick glance over missing data intervals. The radial graph in Suppl. Figure 7 represents a visualization of observed sleeping behaviour. In addition to several other visualization around GARMIN® data, microbiome taxonomy data was also imported for all participants and was visualized to observe microbiome changes of an individual over six timepoints (Suppl. Figure 8). In the long run, the overall idea is to extend this portal where study managers or researchers can visualize all the longitudinal data (activity tracker data, demographics, stool, questionnaires) in one place related to a single study.

### Acquisition of dietary data

Participants were asked to fill in a comprehensive questionnaire regarding their usual diet before the start of the study as well as regular questionnaires regarding the diet within the week before a stool sample was taken.

### Stool sample processing and sequencing

Provided stool samples were processed subsequently as has been described in detail earlier [42]. Briefly, after bead beating of samples, DNA was extracted using the QIAamp DNA fast stool mini kit automated on the QIAcube (Qiagen, Hilden, Germany) according to the manufacturer’s protocol. Blank extraction controls were included at this point and within the following sequencing processes. Three µl of diluted DNA was used for amplification of variable regions V1 and V2 of the 16S rRNA gene using the primer pair 27F-338R in a dual-barcoding approach according to Caporaso et al. [43]. Sequencing was performed on the Illumina MiSeq machine using v3 chemistry for 2 × 300 bp paired-end reads (Illumina Inc., San Diego, CA, USA). Subsequently, samples were demultiplexed with 0 mismatches in the barcode sequences.

### Quantitative real-time PCR *Veillonella atypica*

For samples of elite athletes (n = 12) and of all participants before start of intervention, additional quantitative RT-PCR analysis was performed to confirm findings described by Scheiman et al. [24]. Primers that were used for quantitative RT-PCR analysis have been described elsewhere [24]. Amplification was carried out in a LightCycler®480 instrument (Roche Deutschland Holding GmbH, Grenzach-Wyhlen, Germany) using the SYBR® Green I Mastermix (Roche Deutschland Holding GmbH) according to the manufacturer’s protocol. Absolute quantification of strain abundance was calculated with the LightCycler® 480 Software, Version 1.5 and by using internal standard curves with DNA from those strains purchased by the DSMZ (DSM 20,739, type strain *V. atypica*). The master mix was prepared according to the manufacturers protocol and the following amplification protocol was used: initial denaturation 10 min 95 °C, 30 sec 95 °C and 60 sec 60 °C for 50 cycles and final melting curve analysis from 65 to 95 °C (1°/sec).

### Sequence data processing

Data processing was performed using the DADA2 version 1.10 [44] workflow for big datasets (https://benjjneb.github.io/dada2/bigdata.html) resulting in abundance tables of amplicon sequence variants (ASVs). Briefly, all sequencing runs were handled separately (workflow adjusted for V1-V2 region can be found here: https://github.com/mruehlemann/ikmb_amplicon_processing/blob/master/dada2_16S_workflow.R) and finally collected in a single abundance table per dataset, which underwent chimera filtering. ASVs underwent taxonomic annotation using the Bayesian classifier provided in DADA2 and using the Ribosomal Database Project (RDP) version 16 release. Sample (*n* = 1) with less than 10,000 sequences was not considered for further analysis.

### Statistical analyses of steps, sleep length and diet

Statistical tests were applied to answer two questions about within group variation: (i) “Is there a group-wise change from the start of the study until the end of the physical activity intervention?” and (ii) “Is there a group-wise change towards the end of the intervention period and after it?”. For both questions, the same model design was applied “y ~ participant_id + day”, where y represents the dependent variable in question. Participant identifiers were included in the formula to control for effects of the affiliation of samples. To capture the dynamics of the gut microbiota under physical intervention, models aiming to answer question (i) were applied to data points that were taken from the start of the study until the last day of the physical activity intervention. Models aiming to answer question (ii) were applied to data points that were taken within an interval of 15 days before and 15 days after the last day of intervention. Tests were carried out within each group.

Within group variation in means of daily steps and sleep length was analysed using linear models. Patterns of dietary components were summarized by principal component analysis (PCA) using the R package factoextra version 1.0.5 [45]. Changes in overall dietary pattern were analysed using the first principal component as dependent variable on linear models. Changes in individual dietary components were analysed using linear models on logarithm-transformed data. For the latter, false discovery rate multiple correction was applied. False discovery rate P-value correction in accordance with Benjamini and Hochberg [46] was employed within each test set for the variable “day”. *P*-values were calculated using sequential ANOVA. For P values and adjusted P values, a significance threshold of 5 % was applied. Statistical designs were used as described above.

### Statistical analyses of microbiome data

Statistical tests were applied to answer two questions stated in the previous section (Steps, sleep length and diet analysis). For microbiome data from participants of the groups control, endurance and strength, the following model design was used: “y ~ participant_id + PC1 + day”. Here, the first principal component (PC1) derived from diet questionnaires was included to control for possible effect of diet on the microbiome. The differences between single timepoint microbiome data from the group of elite athletes and physically inactive participants were tested using the model design “y ~ PC1 + group”. Samples from physically inactive participants were selected from the first faecal collection and matched for age and sex.

Microbiome alpha diversities measures, richness and diversity, were estimated using Chao1 and inverse Simpson (InvSimp). These measures were calculated on rarefied ASV table to 10.000 sequences per sample. For each group, the effect of exercise intervention on the alpha diversity (logged units) was tested using linear models. P-values were calculated using sequential ANOVA, with an alpha level of 5 %. Beta diversity was calculated on rarefied ASV table with Bray-Curtis dissimilarity. Nonmetric multidimensional scaling (NMDS) was applied to visualize the level of dissimilarity of samples. Differences in community structure were tested using adonis2, with significance assessed sequentially (by = “term”) with an alpha level of 5 % and after 999 permutations. Rarefaction, calculation of diversity indexes and NMDS were performed with the R package vegan version 2.5-5 [47].

Difference in ASV average abundances was tested using DESeq2 version 1.24.0 using default parameters [48]. Prior testing, ASVs were pre-filtered to be present in at least 10 % of the samples and with a mean of 10 sequences per sample in the rarefied ASV table. This filtering was applied in each set of samples being tested. Library sizes were estimated using the method “poscounts”, which calculates a modified geometric mean by taking the n-th root of the product of the non-zero counts according to DESeq2 manual. For testing the variation in ASV occurrence, same filtering criteria were used. Occurrence was modelled employing binomial linear models. P values for the term “day” were calculated based on Wald test as implemented in the R package survey version 3.1–11 [49]. False discovery rate multiple test correction was applied in each set of tests for both abundance and occurrence models. Adjusted *P* value significance cut off was 0.05.

The differences in the qPCR-based DNA concentration of *Veillonella* in physically inactive and elite athletes (n = 12) was tested using Wilcoxon signed-rank test. All tests and data manipulation were conducted in R version 3.6.2.

### Statistical analyses of biometrics and blood profile

Biometrics and blood profiles were tested in separated batches. Differences of measures from before and after intervention were estimated within each group. These were tested using paired Wilcoxon signed-rank test. Within each group, false discovery rate P value correction was applied. Adjusted P value significance cut-off was 0.05. Rank-biserial correlation was calculated as described in [50]. Difference in participants’ average daily steps, accumulated hours of APHRM and average sleeping hours were compared among groups using Wilcoxon tests and visualized with the R package ggpubr version 0.2 [51].

## Supplementary Information

**Additional file 1.**

## Data Availability

The datasets generated and analysed during the current study are available at the European Nucleotide Archive (ENA, Project accession number: PRJEB38835), please see https://www.ebi.ac.uk/ena/browser/view/PRJEB38835. Codes used for data analyses are available at https://github.com/LucasMS/sport_intervention_2020.
