# Supplementary material for: Short-term physical exercise impacts on the human holobiont obtained by a randomised intervention study
Source: BMC Microbiol. 2021 Jun 2;21:162. doi: 10.1186/s12866-021-02214-1 (PMC8170780; doi:10.1186/s12866-021-02214-1)
Supplement: Supplementary file 1 — Additional file 1. [file 12866_2021_2214_MOESM1_ESM.docx]

# Supplementary Figures


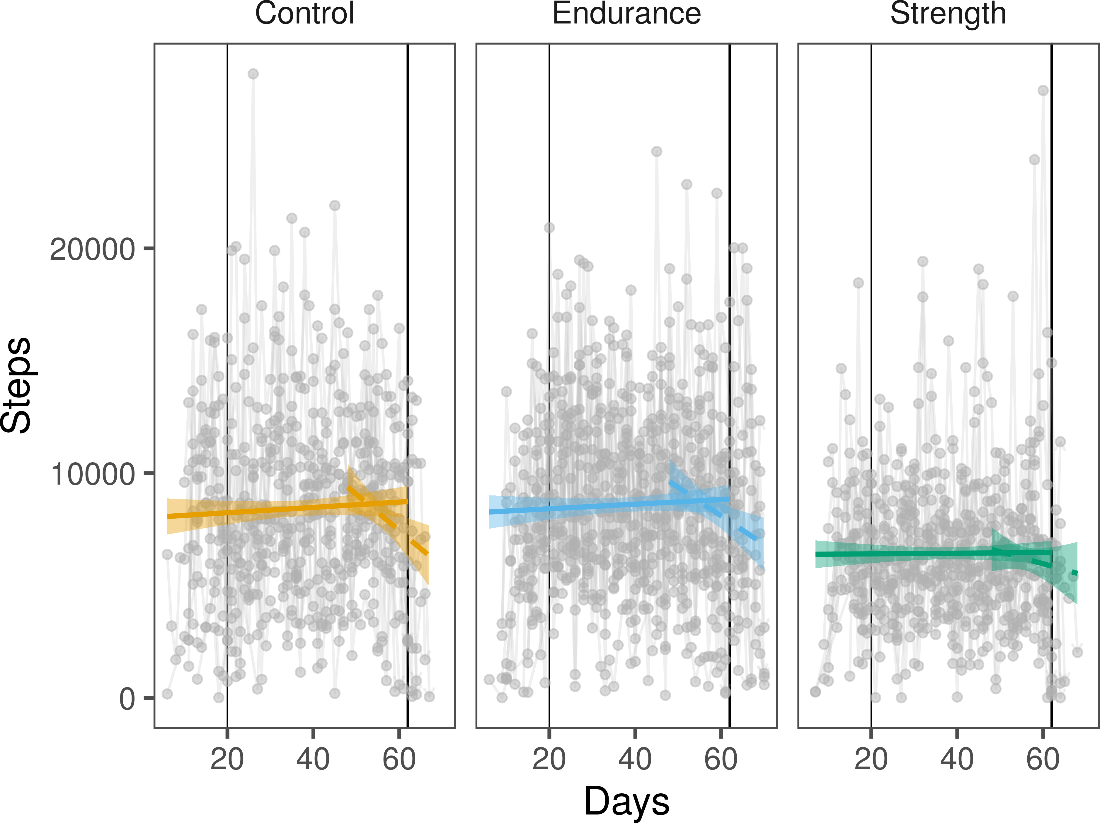


Suppl. Fig. 1: Participant’s daily steps. Participants’ data are connected by line. Two scenarios were modeled to test for within group variation before and during the exercise intervention period (solid line) and to test for within group variation after the exercise intervention (dashed lines). Vertical lines indicate the beginning and end of the exercise intervention interval. No significant variation was found.


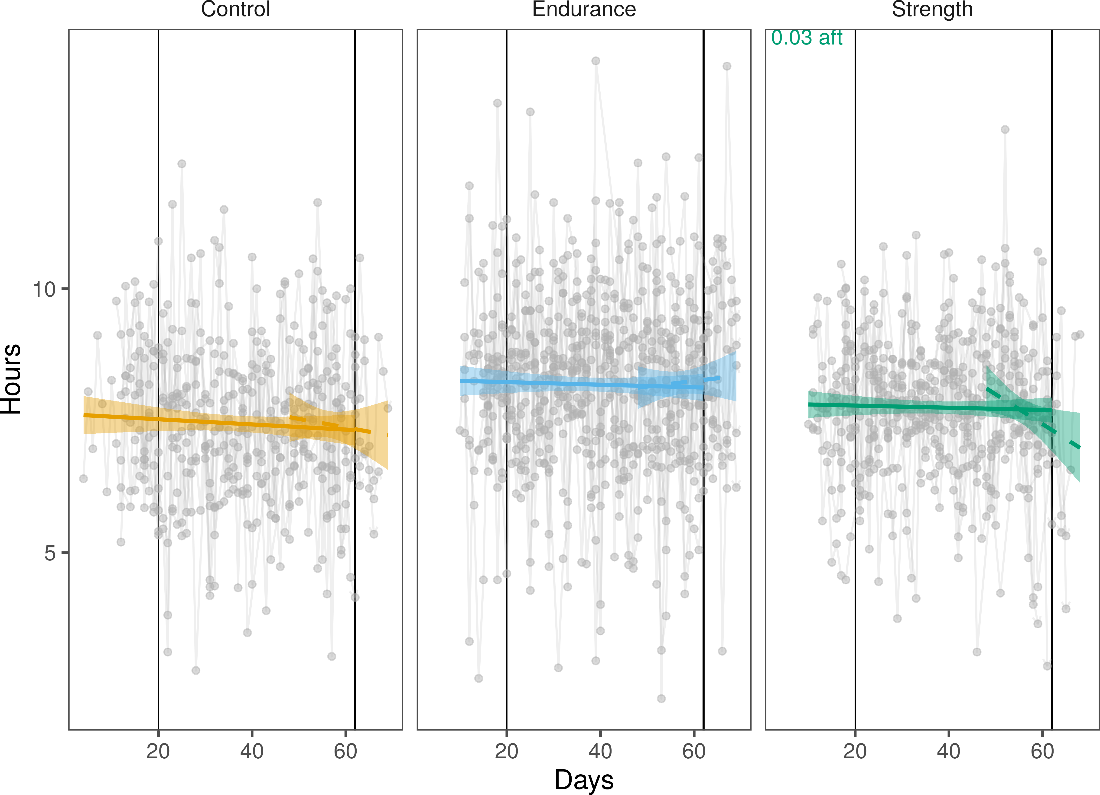


Suppl. Fig. 2: Participant’s sleeping hours. Participants data are connected by line. Two scenarios were modeled to test for within group variation before and during the exercise intervention period (solid line) and to test for within group variation after the exercise intervention (dashed lines). Vertical lines indicate the beginning and end of the exercise intervention interval. P values of significant results (P < 0.05) are shown.


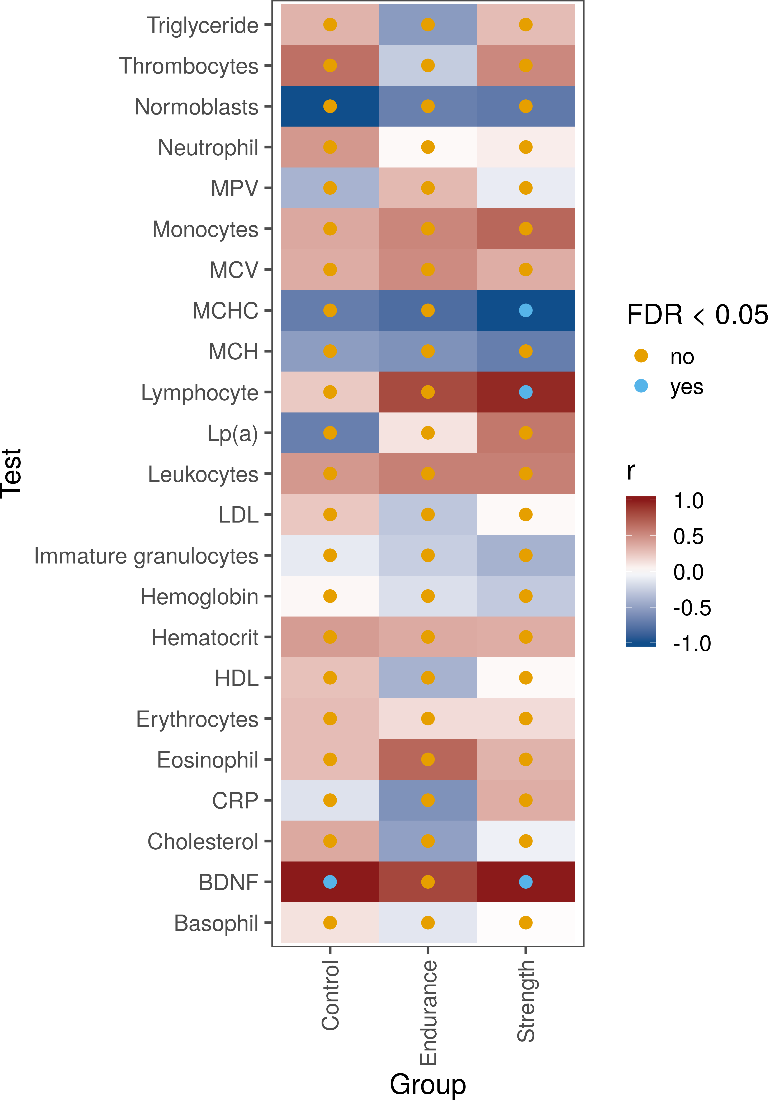


Suppl. Fig. 3: Blood profile changes before and after exercise intervention period. Blood profile changes before and after exercise intervention period. Tests were carried out within each group. False discovery rate P values are coded by colour. Effect sizes were inferred with rank-biserial correlation (r). Names abbreviated are mean platelet volume (MPV), mean corpuscular volume (MCV), mean corpuscular hemoglobin concentration (MCHC), Mean corpuscular haemoglobin (MCH), lipoprotein (a) (Lp(a)), low-density lipoprotein (LDL), high-density lipoprotein (HDL), C-reactive protein (CRP) and Brain-derived neurotrophic factor (BDNF).


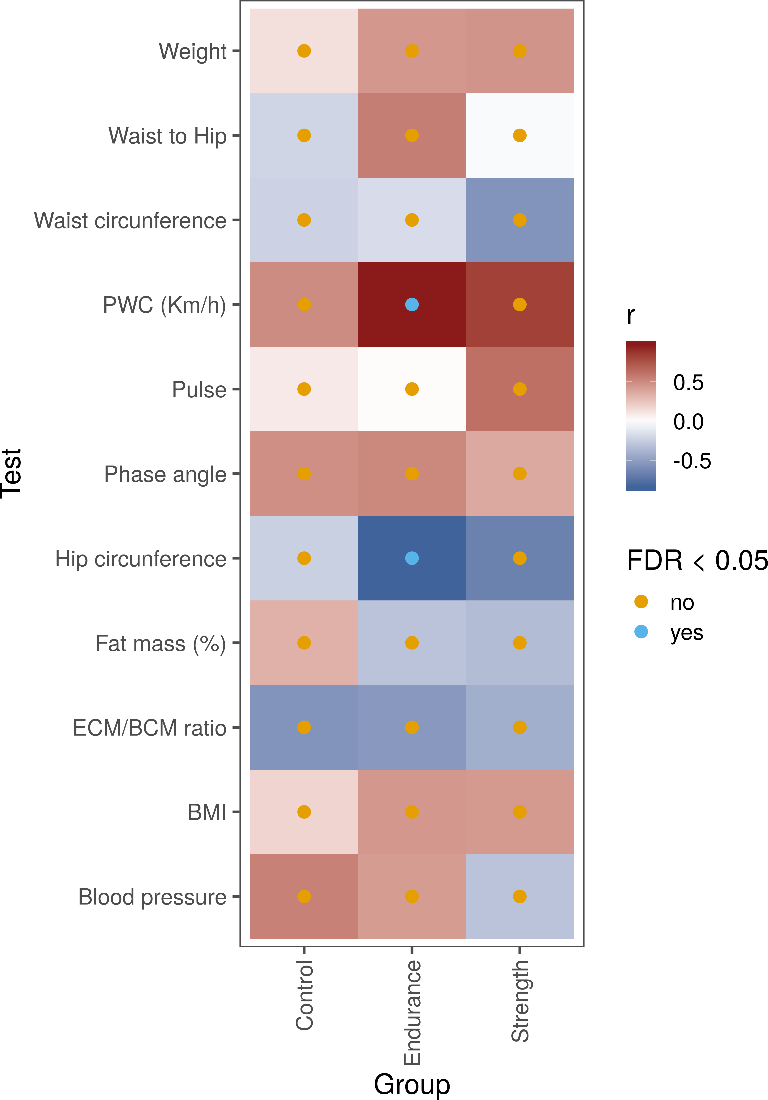


Suppl. Fig. 4: Changes ins body measures and physical working capacity before and after exercise intervention period. Tests were carried out within each group. False discovery rate P values are coded by colour. Effect sizes were inferred with rank-biserial correlation (r). Names abbreviated are physical working capacity (PWC), ratio of extracellular mass to body cell mass (ECM/BCM ratio) and body mass index (BMI).


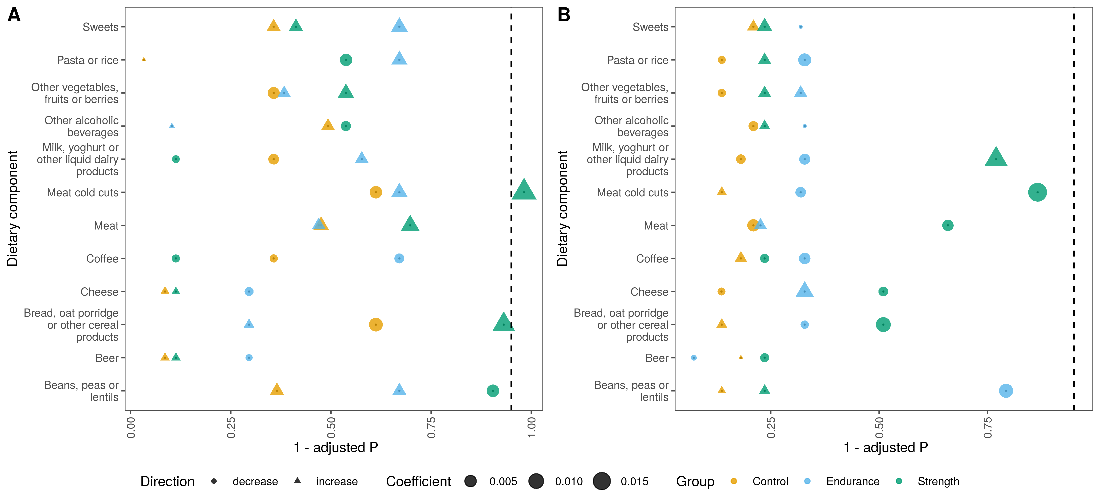


Suppl. Fig. 5: Participant’s dietary component intake. Two scenarios were modeled (A) to test for within group variation before and during the exercise intervention period and (B) to test for within group variation after the exercise intervention. Direction of change was inferred from β coefficients.


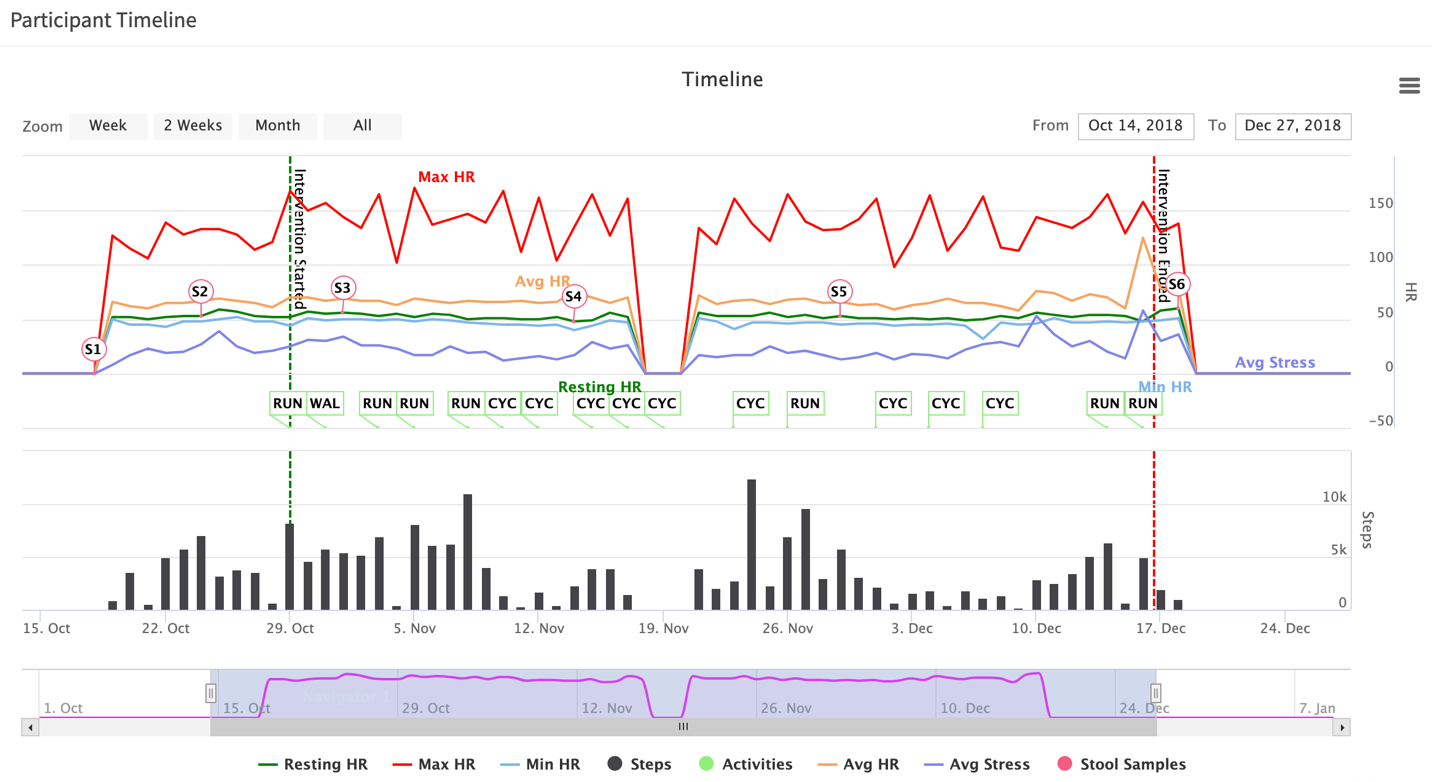


Suppl. Fig. 6: Overall timeline from 14/10/18 till 27/12/18 for a participant. Longitudinal representation of a given participant data from interactive web portal with visible missing data for two dates i.e. 18^th^ and 19^th^ of October when participant was not wearing GARMIN® device. This timeline includes HR, stress, steps and activity data from GARMIN® along with meta data marking intervention and stool sample dates. The study manager can zoom up to 15-minute interval and simply enable or disable any data series to focus.


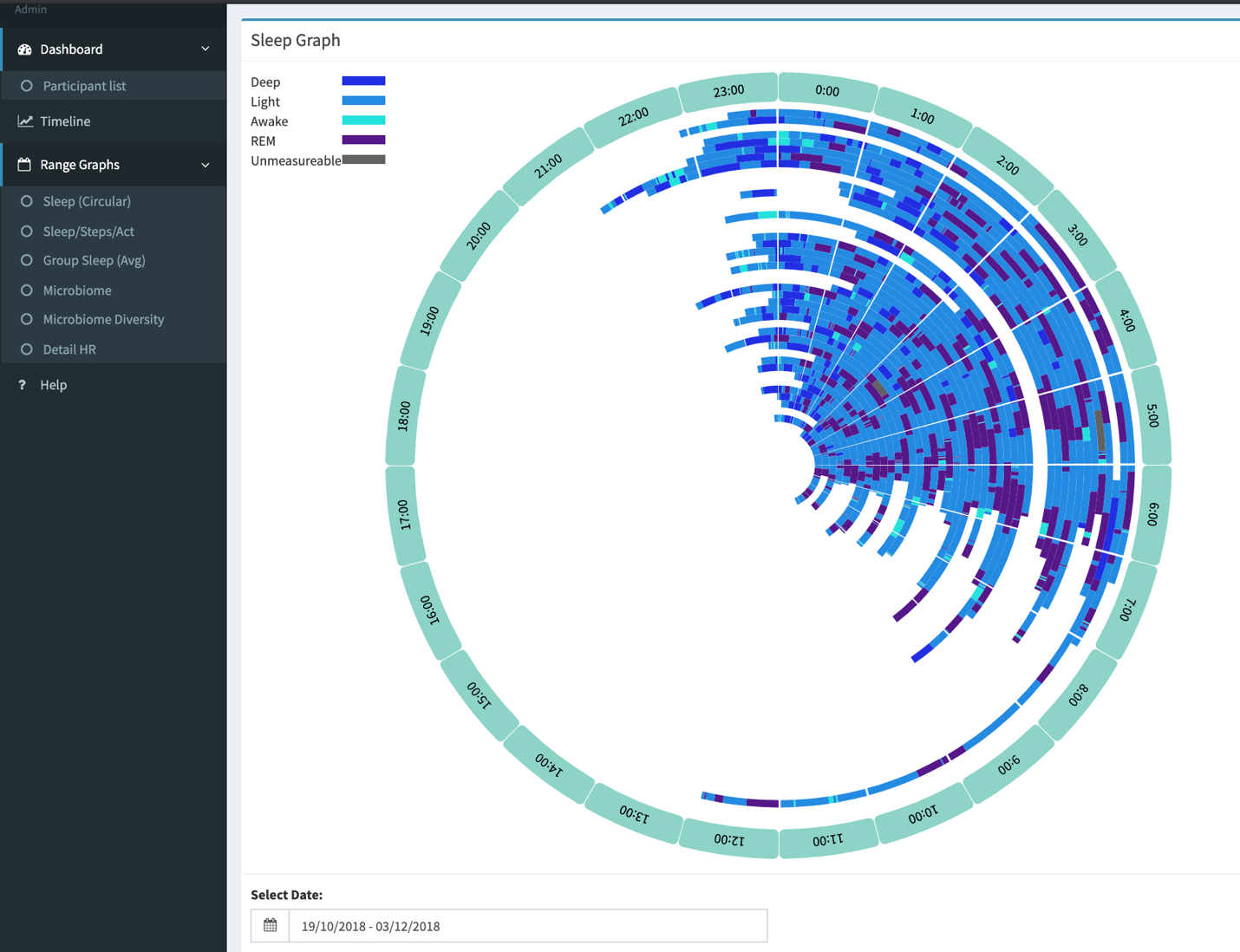


Suppl. Fig. 7: Sleep pattern of 45 nights from 19/10/18 till 03/12/18 for a participant. Each ring presents a day, consists of different sleep phases and timings (date increasing in outward direction). Overview of a participant’s sleep behavior who goes to bed between 22:00-23:00 and wakes up between 7:00-8:00 with an exception on one weekend (01/12/18) waking up at 12:00. This participant is lacking data for 20^th^ and 21^st^ November.


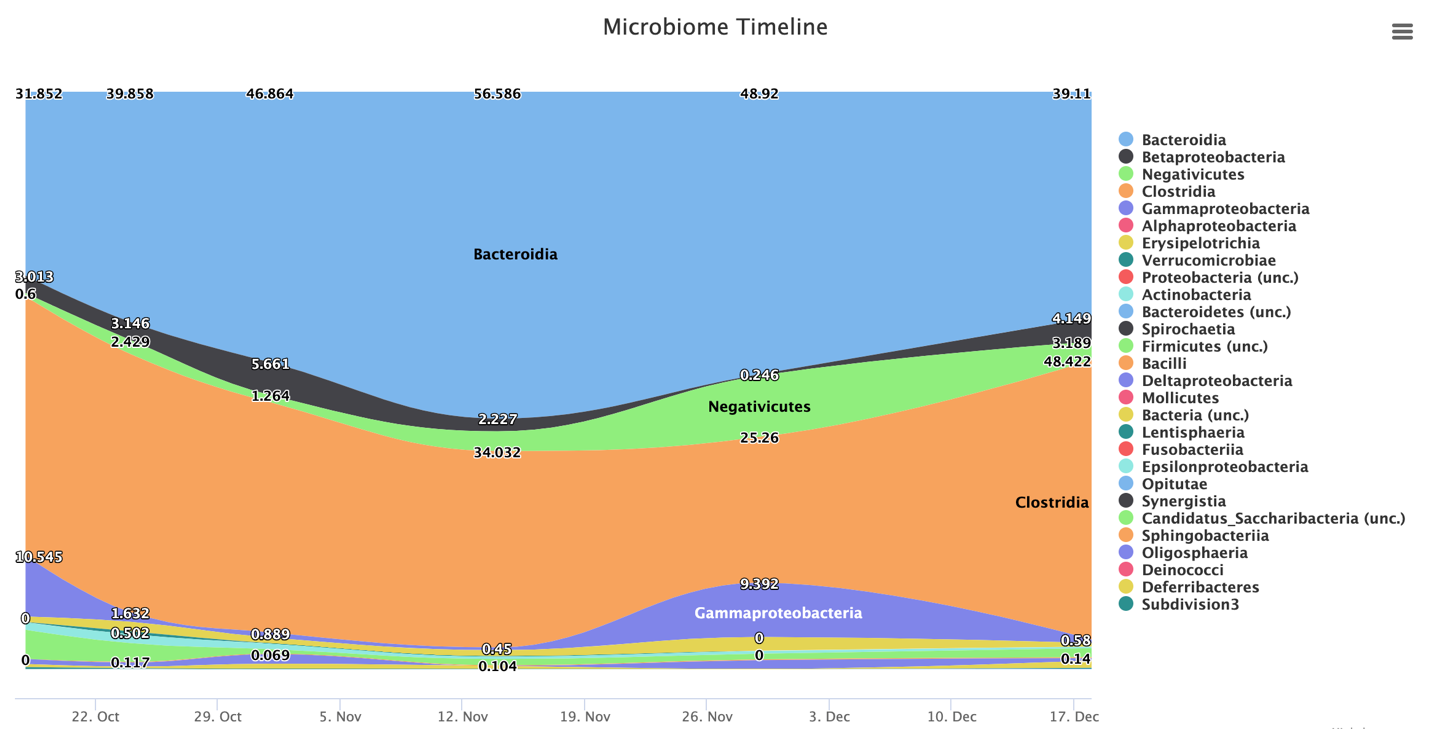


Suppl. Fig. 8: Microbiome sample data of a participant from interactive web portal. Stream graph representation of changes in microbiome (shown is class level) for six stool samples from one study participant. As can be seen, microbiome varies slightly during the study period.
